# Longitudinal Cognitive Trajectories in Older Adults with Restless Legs Syndrome or Willis–Ekbom Disease

**DOI:** 10.3390/life14040430

**Published:** 2024-03-24

**Authors:** Ioannis Liampas, Vasileios Siokas, Andreas Kyrozis, George Sakoutis, Mary Yannakoulia, Mary H. Kosmidis, Paraskevi Sakka, Nikolaos Scarmeas, Georgios M. Hadjigeorgiou, Efthimios Dardiotis

**Affiliations:** 1 Department of Neurology, University Hospital of Larissa, School of Medicine, University of Thessaly, 41100 Larissa, Greece; bill_s1983@hotmail.com (V.S.); gsakoutis@gmail.com (G.S.); hadjigeorgiou.georgios@ucy.ac.cy (G.M.H.); edar@med.uth.gr (E.D.); 2 1st Department of Neurology, Aiginition Hospital, National and Kapodistrian University of Athens Medical School, 11528 Athens, Greece; akyrozis@med.uoa.gr (A.K.); ns257@cumc.columbia.edu (N.S.); 3 Department of Nutrition and Dietetics, Harokopio University, 17671 Athens, Greece; 4 Laboratory of Cognitive Neuroscience, School of Psychology, Aristotle University of Thessaloniki, 54124 Thessaloniki, Greece; kosmidis@psy.auth.gr; 5 Athens Association of Alzheimer’s Disease and Related Disorders, 11636 Marousi, Greece; vsakka@ath.forthnet.gr; 6 Taub Institute for Research in Alzheimer’s Disease and the Aging Brain, The Gertrude H. Sergievsky Center, Department of Neurology, Columbia University, New York, NY 10032, USA; 7 Department of Neurology, Medical School, University of Cyprus, 2408 Nicosia, Cyprus

**Keywords:** memory, language, attention, executive function, visuospatial function

## Abstract

**Background**: Restless legs syndrome/Willis–Ekbom disease (RLS/WED) has occasionally but not consistently been associated with cognitive and most notably language and executive impairment. The present study was conducted to investigate the cognitive trajectories of older individuals with RLS/WED. **Methods**: Participants were drawn from the randomly selected, older (>64 years), population-based HELIAD cohort. Individuals without dementia and with available neuropsychological evaluations at baseline and follow-up were considered for potential eligibility. A comprehensive assessment examining five principal components of cognition (memory, visuo-spatial ability, attention, executive function, and language) was administered to the participants. Generalized estimating equation analyses were used to examine the unadjusted and adjusted (for critical factors and covariates) effects of RLS/WED on cognition over time. **Results**: A total of 1003 predominantly female (59.5%), older (72.9 ± 4.9 years) participants with follow-up evaluations after a mean of 3.09 ± 0.85 years and without dementia at baseline and follow-up were included in the present study. Among them, 81 were diagnosed with RLS/WED at baseline. Global cognition, memory, attention, and executive and visuo-perceptual skills did not differ between those with and without RLS/WED. However, the RLS/WED group performed worse on language at baseline by a standard deviation of 0.249, while demonstrating a mitigated language decline over time, by a standard deviation of 0.063. The unadjusted models yielded similar results. **Conclusions**: Our findings were indicative of a baseline language disadvantage among older individuals with RLS/WED, but the initial discrepancy tends to dissolve over time.

## 1. Introduction

Restless legs syndrome/Willis–Ekbom disease (RLS/WED) is a sensorimotor network disorder with an elusive underlying pathophysiology [1,2,3]. Despite being generally considered a “benign” entity, RLS/WED has been related to sleep and emotional disorders that may affect quality-of-life measures deleteriously [4,5,6,7]. Previous research has occasionally associated RLS/WED with cognitive impairment as well. This relationship is theorized to be mediated by comorbid sleep and affective disturbances [8]. However, relevant evidence is scarce and contradictory, with sporadic reports even suggesting that individuals with RLS/WED outperform normal controls in several neuropsychological domains [8]. 

Pearson and colleagues were the first to investigate cognitive impairment in RLS/WED. They reported that middle-aged and older adults with RLS/WED recorded poorer performance in pre-frontally mediated functions, namely verbal fluency, comparably to that anticipated after one night of sleep deprivation [9]. However, no differences were found in less frontally dependent and global cognitive measures. Subsequent studies reproduced these findings in middle-aged and older individuals, indicating that language, executive function (and most notably verbal fluency), and, less frequently, attention may be impaired in the course of RLS/WED [10,11,12,13,14]. These findings have even been replicated after adjusting for multiple potentially important confounders, including sleep duration and quality and anxiety and depression scores, as well as hypnotic and antidepressant medication intake, suggesting that RLS/WED may directly predispose the individual to cognitive dysfunction [10,14]. Previous research has also indicated (although less consistently) that visuo-perceptual skills may be affected in the course of RLS/WED [14]. Of note, Galbiati and colleagues showed that middle-aged subjects with severe RLS/WED presented generalized cognitive deficits compared to healthy controls (involving the domains of executive function and attention, working and long-term memory, and visuo-perceptual skills) that responded quite well to the administration of dopamine agonists [15]. Finally, RLS/WED individuals with concomitant Parkinson’s disease (PD) or multiple sclerosis (MS) have exhibited greater global cognitive deficits and perceived impairments in comparison with PD or MS controls without comorbid RLS/WED [16,17], further supporting the direct effect of RLS/WED on cognition.

On the other hand, previous conflicting reports (the largest study to date included) concluded that RLS/WED was not associated with cognitive (neither executive nor attentional) dysfunction in older adults [18,19,20]. There is even scarce evidence that RLS/WED subjects may outperform normal controls in measures of verbal memory and executive function [21]. Intriguingly, Gamaldo and colleagues compared individuals with RLS/WED and partially sleep-deprived controls in terms of global intelligence and executive function and found a superior performance of the former group, proposing a relative degree of sleep loss adaptation in individuals with RLS/WED [22].

Overall, there is considerable controversy regarding the existence of cognitive impairment, as well as the domains of cognition affected in individuals with RLS/WED. Of note, even among consistent reports, the direct or indirect impact of RLS/WED on cognition is a matter of substantial controversy. The majority of current evidence stems from relatively small-sized samples, while important confounders have only been controlled occasionally. Most importantly, there is a remarkable lack of longitudinal evidence, with only cross-sectional and case–control articles published to date. Therefore, the aim of undertaking the present study was to investigate the cognitive trajectories of patients with RLS/WED in relation to those without RLS/WED using a large, prospective, population-based cohort of older individuals. 

## 2. Materials and Methods

### 2.1. Study Design, Participants, and Settings

The present article adheres to the STROBE reporting guidelines (Strengthening the Reporting of Observational Studies in Epidemiology) [23]. Our sample was drawn from the Hellenic Epidemiological Longitudinal Investigation of Aging and Diet (HELIAD). The rationale and key elements of the HELIAD study have been previously described in great detail [24]. In brief, the HELIAD is a multidisciplinary, population-based, prospective cohort primarily investigating the descriptive and analytical epidemiology of dementia and cognitive impairment in the older Greek population. The Institutional Ethics Review Boards of the University of Thessaly and the National and Kapodistrian University of Athens approved all procedures prior to the initiation of the study. Informed consent was acquired from all participants or surrogates prior to participation.

Participant selection was performed through random sampling from the rosters of the older individuals (≥65 years) of two Greek municipalities, Larissa (province of Thessaly) and Marousi (metropolitan city of Athens). Extensive collaborative assessments designated by a consortium of expert neurologists, neuropsychologists, and dieticians were conducted during baseline and follow-up. Relevant information was collected from participants or participant carers (first-degree relatives, etc.) whenever deemed necessary. 

For the present analysis, eligible individuals were not diagnosed with dementia at baseline (as even mild to moderate dementia may interfere with the proper diagnosis of RLS/WED) and follow-up (to exclude the potential impact of underlying neurodegenerative processes on cognitive trajectories). Moreover, participants had conclusive baseline diagnostic data on RLS/WED and available neuropsychological assessments at both visits. 

### 2.2. Neuropsychological Assessments and Diagnostic Approach

Cognition was evaluated by trained neuropsychologists according to a designated approach involving the comprehensive assessment of all major cognitive domains [24,25,26]. Comprehensive assessments took place both at baseline and at follow-up. A brief screening of global cognition and orientation was performed using the mini-mental state examination (MMSE), and a gross estimate of the intellectual level of the participants was acquired using a Greek multiple-choice vocabulary test. Episodic memory was assessed through the Greek verbal learning test (verbal memory) and the Medical College of Georgia Complex Figure Test (MCG; non-verbal memory); language was assessed based on the semantic and phonological fluency tasks and subtests of the Greek version of the Boston Diagnostic Aphasia Examination short form (the Boston Naming Test—short form; and selected items from the Complex Ideational Material Subtest to assess verbal comprehension and repetition of words and phrases); visuospatial ability was assessed based on the Judgment of Line Orientation abbreviated form, the MCG copy condition, and the clock-drawing test; attention and processing speed were assessed based on the Trail Making Test-Part A (TMT-A); executive functioning was assessed based on the Trail Making Test-Part B (TMT-B), Anomalous Sentence Repetition (created for the present investigation), and Graphical Sequence Test and Motor Programming. Individual raw test scores were converted into z-scores using the mean and SD values of the cognitively normal (without dementia or MCI) participants at baseline. Higher scores were consistent with better cognitive performance.

The diagnostic classification of the participants according to their cognitive status was established during expert consensus meetings involving senior neurologists and neuropsychologists. Dementia and AD were diagnosed according to the Diagnostic and Statistical Manual of Mental Disorders-IV-text revision criteria [27] and the National Institute of Neurological and Communicative Disorders and Stroke/Alzheimer Disease and Related Disorders Association criteria [28], respectively. The diagnosis of vascular dementia was based on history or clinical evidence of stroke, the presence of a clear temporal relation between stroke and the onset of dementia, and the results of the Hachinski Ischemia Scale score [29]. Lewy body and frontotemporal dementias were considered according to respective criteria [30,31]. Particular focus was placed on identifying potential comorbidities that could affect cognitive performance through screening the participants for depression, anxiety, essential tremor, behavioural symptoms, Parkinson’s disease, and a personal history of cerebrovascular disease accounting for the onset or deterioration of cognitive decline [32,33]. Information was also gathered on self- and carer-reported comorbidities, regular medication intake, sleep and dietary habits, mental and physical activity, and laboratory tests (imaging studies and blood examinations) when available [33,34]. 

A detailed description of the diagnosis of RLS/WED and other neuropsychiatric conditions and the detailed definitions of all potential confounders are provided in our previous report focusing on the descriptive and analytical epidemiology of RLS/WED in the older Greek population [34]. It is highlighted that RLS/WED was diagnosed according to the 2003 revision of the 1995 International RLS Study Group clinical diagnostic criteria [35].

### 2.3. Statistical Analysis

Our previous report focusing on the descriptive and analytical epidemiology of RLS/WED in the older Greek population found that age; biological sex; anxiety levels (according to the 22-point Hospital Anxiety and Depression Scale); sleep quality (poor, regular, and good; based on the Sleep Index II from Medical Outcomes Study); history of traumatic brain injury (TBI); and dietary parameters, i.e., Mediterranean diet—MeDi-score (55-point MeDi scale)—and daily energy intake (quantified using the food frequency questionnaire; participants were classified into four categories using mean and SD values: low–low/moderate–moderate/high–high) were related to the presence of RLS/WED in our sample [34]. On the other hand, body mass index, educational attainment, depression levels, occupational history, socioeconomic status, smoking, alcohol consumption, caffeine intake, anti-depressive medication intake, risk of malnutrition, physical activity, sleep duration, and comorbidities other than TBI were not associated with RLS/WED in our cohort [34].

The longitudinal cognitive trajectories of individuals with and without RLS/WED were compared using generalized estimating equation (GEE) analyses. GEE accounts for the potential correlation of repeated measurements in the same individual. We treated each participant’s baseline and follow-up evaluations as a cluster. Exchangeable (compound symmetry) covariance matrices were conventionally chosen as working correlation structures. Six consecutive GEE models were explored using composite (one) and individual domain (five) cognitive measurements as the dependent scale variables. Preliminary unadjusted analyses featuring the main effects of RLS/WED diagnosis and time from baseline, as well as the RLS/WED by time interaction terms, were performed. Subsequently, adjusted models additionally incorporating the main effects of age, sex, anxiety levels, sleep quality, history of TBI, MeDi scores, and daily energy intake were tested. 

All statistical analyses were performed using the IBM SPSS Statistics Software Version 26 (Chicago, IL, USA). The corrected α = 0.01 cut-off (for 5 comparisons) was used in individual domain analyses. Baseline differences between those with and without RLS/WED were explored using an unpaired *t*-test and Pearson’s chi-squared test. 

## 3. Results

### 3.1. Baseline Characteristics and Missing Data

The HELIAD cohort consisted of 1984 participants at baseline. Among them, follow-up data were available for 1105 individuals. Thirty participants were subsequently excluded from the present analysis due to a diagnosis of dementia at baseline (*n* = 28) or an inconclusive baseline cognitive diagnosis (*n* = 2). An additional 67 participants were excluded because they converted to dementia at follow-up (*n* = 65) or had an inconclusive follow-up cognitive diagnosis (*n* = 2). Finally, 1010 participants with available baseline and follow-up assessments and without dementia (at baseline or follow-up) were assembled. Among them, seven participants were excluded on the grounds of an inconclusive RLS/WED diagnosis (due to missing data), leaving a total of 1003 participants with follow-up evaluations after a mean of 3.09 years (standard deviation = 0.85) and 81 with and 922 without RLS/WED at baseline. 

The baseline characteristics of our sample according to the presence or absence of RLS/WED are provided in Table 1. The RLS/WED group included more female participants, exhibited a poorer quality of sleep, and reported a history of TBI more frequently compared to those without RLS/WED. Moreover, individuals with RLS/WED presented greater levels of anxiety and lower daily energy intake than participants without RLS/WED. In terms of cognition, those without RLS/WED performed better on the global index, a difference that was mainly driven by the composite language index.

### 3.2. Differences in Cognition and Cognitive Trajectories in Participants with and without RLS/WED

**Figure 1:**
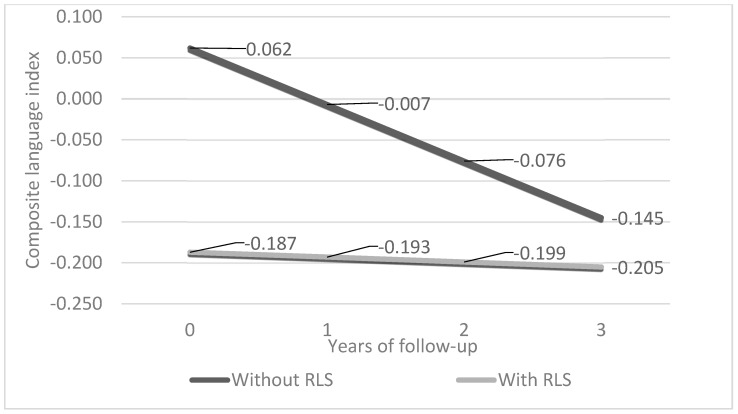
Adjusted GEE (generalized estimating equations) predicted longitudinal trajectories of the composite language index in older adults with and without restless legs syndrome (RLS). Footnote: The GEE model was estimated using exchangeable covariance structure and was adjusted for the mean age (~73 years); anxiety levels (~3 on the Hospital Anxiety and Depression Scale); Mediterranean diet score (~34 on the Mediterranean diet scale) of our sample; the average effects of sex and daily energy intake; a regular–moderate quality of sleep; and a personal history free of traumatic brain injury. The presented illustration assumed 3 years of follow-up (mean follow-up of our sample).

The main effects of RLS/WED and time on cognition, as well as the RLS/WED by time interaction terms, are presented in Table 2. Baseline differences (main effects) and longitudinal associations (RLS/WED by time interactions) between RLS/WED and global cognition, memory, executive function, attention, and visuo-perceptual skills were insignificant. However, baseline language performance and longitudinal trajectories differed between RLS/WED and non-RLS/WED individuals. In particular, the GEE approach (according to the adjusted model) revealed that the RLS/WED group class performed worse on language at baseline by an SD of 0.249. On the other hand, the non-RLS/WED class underwent a steeper (by a yearly SD of 0.063) language decline compared to the RLS/WED group over time (aging effect). Overall, these findings were indicative of distinct regression curves that are inclined to converge over time (due to the better baseline performance and more prominent decline in participants without RLS/WED) (Figure 1).

## 4. Discussion

The present study, to our knowledge, is the first to investigate the longitudinal cognitive trajectories of individuals with RLS/WED in comparison with those without this diagnosis. The results obtained were indicative of a baseline language impairment in the RLS/WED group, which, however, remained steady over time, while the non-RLS/WED group showed a gradual decline from baseline to follow-up. On the other hand, global cognition, memory, attention, and executive and visuo-perceptual function did not differ between the two groups. These findings were replicated using both unadjusted and adjusted analyses, suggesting that the potential confounders incorporated in the adjusted model (which were previously associated with RLS/WED in our sample) did not exert a crucial effect on the different language trajectories of individuals with and without RLS/WED.

Previous research has revealed that individuals with RLS/WED may exhibit relative cognitive deficits in frontally dependent functions, language, and executive function [9,10,12,15]. Language itself is heavily dependent on executive skills, especially in older adults [36,37,38,39]. Frontally mediated executive mechanisms have been suggested to coordinate and monitor linguistic tasks and most notably verbal fluency. Executive function is crucial for the initiation of word production and the retrieval of appropriate words, the preservation of flexible thinking, and the temporary retention of important information while inhibiting inappropriate and previously generated responses [40]. Herein, researchers often incorporate linguistic tasks (most often verbal fluency) into executive assessments. Irrespective of the clustering strategies of neuropsychological evaluations, published cross-sectional reports suggest that in the presence of RLS/WED, language and executive function are most consistently impaired [9,10,12,15]. On the other hand, the remaining cognitive domains, such as memory, visuospatial perception, and attention or processing speed, were seldom found to be impaired among individuals with RLS/WED.

However, there is also a considerable body of cross-sectional evidence stipulating that no cognitive function differentiates those with RLS/WED from those without [18,19,20]. To date, the contradictions among published articles have been mainly attributed to methodological considerations such as heterogeneous study designs (e.g., outpatient clinic-derived vs. population-based samples) and a lack of matching for key parameters that often remained unaccounted for in the statistical plan as well, e.g., severity of RLS/WED, pharmacological management, and so on. Of note, one research group has reported that although patients with RLS/WED perform worse than non-RLS/WED controls on verbal fluency tasks, they outperform recently sleep-deprived controls (according to 14-day sleep restriction protocols) [9,22]. Therefore, it was speculated that sleep deprivation may be the underlying cause of cognitive impairment, and an adaptive response to sleep loss over time might explain the cognitive advantage of RLS/WED vs. sleep-deprived individuals. Similarly, our findings could also reflect an adaptive behaviour of individuals with RLS/WED over time. This adaptation could lead to the relative “floor effect” visualized in Figure 1, which counterbalances at least part of the initial worse language performance in the RLS/WED group. Hence, the poorer baseline language performance of those with RLS/WED was followed by an attenuated course of decline (incorporating aging and adapting effects), and the cognitive trajectories of individuals with and without RLS/WED tended to converge at follow-up, dissolving any baseline differences. 

On the other hand, our findings may have nothing to do with the long-term adaptation of individuals with RLS/WED but rather with the management of their condition and comorbid entities. Intriguingly, results reported by Galbiati and colleagues may provide an alternative explanation for the convergence of language trajectories at follow-up [15]. In particular, the aforementioned study found that the pharmaceutical management of RLS/WED (with dopamine agonists) could improve relative cognitive deficits to “normal” levels. However, none of the 91 participants in the present analysis reported receiving treatment with dopamine agonists at baseline, while only 2 participants reported dopamine agonist intake at follow-up; therefore, this storyline is rather improbable. Of course, medicinal and non-pharmaceutical interventions addressing sleep and affective disorders (as well as other RLS/WED-relevant parameters) may have also taken place throughout the follow-up and may account for the coming together of language trajectories [41,42,43]. Notably, participation in an epidemiological study may alert participants and their informants to several health aspects that previously went unnoticed [44]. The perplexing nature and labyrinthine network of such interactions should be addressed in future analyses that will document potential confounders and mediators in detail, as well as relevant interventions throughout the follow-up.

Finally, it is theoretically plausible that our findings are a result of methodological shortcomings or chance (the RLS/WED–language association at baseline may represent a type I statistical error/the loss of such association at follow-up may represent a type II error). The strong statistical associations (especially regarding the time-interaction product) argue against the latter scenario. However, the former scenario cannot be disqualified with certainty. RLS/WED-dependent modifiers of language that were not recorded in the context of HELIAD (e.g., mediators such as RLS/WED severity) or RLS/WED-independent determinants that were more prominent at follow-up (unaccounted for in our analyses) may assume a role in the dwindling of language differences between RLS/WED and non-RLS/WED participants over time. 

### 4.1. Strengths and Limitations 

The present study was based on a large, randomly selected, population-based cohort of older individuals. The multidisciplinary, collaborative planning of the HELIAD study by a panel of expert neurologists, neuropsychologists, and dieticians allowed the documentation of numerous parameters and their inclusion in our analyses. Thus, the present study included crucial factors and covariates associated with the presence of RLS/WED in our sample [33]. Finally, the prospective design of our cohort allowed us to perform longitudinal investigations, providing several innovative findings that may at least partly explain the contradictory findings of earlier studies on cognitive performance in individuals with RLS/WED. 

On the other hand, our study had several important limitations as well. First, notwithstanding the random selection process, non-response and attrition biases were present [45]. Moreover, despite accounting for a large number of potential confounders, residual confounding may yet exist [46]. For instance, a medication that may interfere with cognition (i.e., anticholinergics, opioids, antihistamines, and so on) or nutrient deficits related to both RLS/WED and reversible cognitive impairment (serum ferritin levels, serum vitamin B12, and folate) were unaccounted for in the present analysis. Furthermore, due to the initiation of our study prior to the introduction of the last revised criteria for the diagnosis of RLS/WED, the 2003 revision of the International RLS Study Group diagnostic criteria was implemented [35,47,48]. As previously mentioned, the use of the updated criteria would potentially establish a more accurate diagnosis, excluding RLS/WED mimics and limiting misclassification bias. In addition to the above, electrophysiological assessments or polysomnography were not available to support the clinically based diagnosis of the disorder [49,50]. Also, our standardized data collection forms did not include any information on the severity of RLS/WED or its official medical diagnosis and treatment. However, none of the 91 participants in the present analysis reported receiving treatment with dopamine agonists at baseline (only 2 at follow-up), suggesting that the diagnosed cases most probably had tolerable symptoms. Finally, a fraction of the recorded parameters were evaluated according to participants’ reporting; therefore, the presence of information bias cannot be ruled out.

### 4.2. Conclusions

We report that individuals with RLS/WED may perform worse on language than those without this condition, but differences tend to dissolve over time. However, we failed to reveal the causation underlying this association. Future research may fill in this literature gap by recording potential confounders and mediators such as RLS/WED severity; sleep disorders; neuropsychiatric comorbidities; targeted treatments; lifestyle or other non-pharmaceutical interventions; detailed medication intake—especially with respect to drugs that may interfere with cognition; and nutrient deficits—especially those related to reversible cognitive impairment. Moreover, focus should be placed on the prospective and longitudinal documentation of this information. Mediation analyses may be performed to quantify the extent to which each of these factors exhibits a causal or casual relationship. 

## Figures and Tables

**Table 1:** Participant characteristics.

Parameter	Without RLS/WED (*n* = 922)	With RLS/WED (*n* = 81)	*p*-Value
Age at baseline (N = 1003)	72.9 ± 5.0	73.3 ± 4.1	0.496
Sex (women/men) (Ν = 1003)	534/388 (57.9/42.1%)	63/18 (77.8/22.2%)	<0.001
Sleep quality (poor/moderate/good) (Ν = 974)	265/272/358 (29.6/30.4/40.0%)	30/37/12 (38.0/46.8/15.2%)	<0.001
Anxiety (0–22-point scale) (N = 1003)	2.3 ± 3.5	4.1 ± 4.7	<0.001
MeDi score (0–55-point scale) (N = 980)	33.8 ± 4.6	33.7 ± 4.4	0.972
Daily energy intake (low–low/moderate-moderate/high-high) (N = 973)	125/355/281/134 (14.0/39.7/31.4/15.0%)	21/25/22/10 (26.9/32.1/28.2/12.8%)	0.023
History of TBI (Yes/No) (N = 986)	96/810 (10.6/89.4%)	19/61 (23.4/76.6%)	0.001
Global cognition (N = 992)	−0.01 ± 0.70	−0.18 ± 0.72	0.040
Memory (N = 984)	0.03 ± 0.85	−0.10 ± 0.82	0.208
Executive function (N = 989)	−0.02 ± 0.73	−0.16 ± 0.67	0.094
Visuospatial skills (N = 979)	0.02 ± 0.79	−0.07 ± 0.90	0.315
Language (N = 990)	0.04 ± 0.81	−0.24 ± 0.81	0.003
Attention (N = 949)	−0.06 ± 1.01	−0.29 ± 1.19	0.072

Footnote: N: number of participants with available data per parameter; n: number of total participants per group; continuous variables are presented as mean ± SD; categorical data are presented as absolute frequencies (percentages); *p*-value refers to differences between the restless legs syndrome/Willis–Ekbom disease (RLS/WED) and non-RLS/WED groups; MeDi: Mediterranean Diet; TBI: traumatic brain injury.

**Table 2:** Cognitive trajectories of patients with RLS/WED compared to non-RLS/WED individuals.

Parameter	Main Effect of RLS/WED (β, 95% CI, *p*-Value)	Main Effect of Time (β, 95% CI, *p*-Value)	Time by RLS/WED Interaction (β, 95% CI, *p*-Value)
Global cognitive score (unadjusted)	−0.157 (−0.320, 0.002), 0.058	**−0.076 (−0.085, −0.067), <0.001**	0.017 (−0.007, 0.041), 0.171
Global cognitive score (adjusted)	−0.128 (−0.294, 0.038), 0.130	**−0.077 (−0.087, −0.068), <0.001**	0.018 (−0.007, 0.045), 0.153
Memory (unadjusted)	−0.118 (−0.302, −0.067), 0.213	**−0.070 (−0.083, −0.057), <0.001**	0.045 (0.002, 0.088), 0.040
Memory (adjusted)	−0.135 (−0.321, 0.051), 0.156	**−0.068 (−0.081, −0.055), <0.001**	0.037 (−0.009, 0.082), 0.114
Visuospatial (unadjusted)	−0.080 (−0.277, 0.117), 0.426	**−0.106 (−0.123, −0.089), <0.001**	0.003 (−0.048, 0.054), 0.907
Visuospatial (adjusted)	−0.033 (−0.235, 0.169), 0.748	**−0.111 (−0.129, −0.094), <0.001**	0.001 (−0.051, 0.054), 0.958
Executive (unadjusted)	−0.148 (−0.306, 0.010), 0.067	**−0.050 (−0.060, −0.040), <0.001**	−0.008 (−0.045, 0.030), 0.695
Executive (adjusted)	−0.100, (−0.258, 0.059), 0.217	**−0.050 (−0.061, −0.040), <0.001**	−0.010, (−0.049, 0.030), 0.630
Language (unadjusted)	**−0.257 (−0.442, −0.073), 0.006**	**−0.066 (−0.077, −0.055), <0.001**	**0.054 (0.016, 0.091), 0.005**
Language (adjusted)	**−0.249 (−0.442, −0.055), 0.012**	**−0.069 (−0.080, −0.058), <0.001**	**0.063, (0.024, 0.103), 0.002**
Attention (unadjusted)	−0.240 (−0.518, 0.038), 0.091	**−0.065 (−0.084, −0.046), <0.001**	−0.010 (−0.071, 0.051), 0.745
Attention (adjusted)	−0.159 (−0.447, 0.130), 0.281	**−0.067 (−0.087, −0.047), <0.001**	0.001 (−0.063, 0.065), 0.971

Footnote: RLS/WED; restless legs syndrome/Willis–Ekbom disease; β: regression coefficient; CI: confidence interval; **bold** denotes statistical significance.

## Data Availability

The data that support the findings of this study are available from the corresponding author upon reasonable request.
